# Anti-SARS-CoV-2 Neutralizing Responses in Various Populations: Use of a Rapid Surrogate Lateral Flow Assay and Correlations with Anti-RBD Antibody Levels

**DOI:** 10.3390/life14070791

**Published:** 2024-06-22

**Authors:** Joël Gozlan, Audrey Baron, Anders Boyd, Maud Salmona, Djeneba Fofana, Marine Minier, Audrey Gabassi, Laurence Morand-Joubert, Constance Delaugerre, Sarah Maylin

**Affiliations:** 1 AP-HP, Saint Antoine Hospital, Laboratory of Virology, 75012 Paris, France; joel.gozlan@aphp.fr (J.G.); djeneba.fofana@aphp.fr (D.F.); laurence.morand-joubert@aphp.fr (L.M.-J.); 2 Reseach’s Department of Saint-Antoine, “Cancer Biology and Therapeutics”, University of Sorbonne, INSERM, 75012 Paris, France; 3 AP-HP, Saint Louis Hospital, Laboratory of Virology, 75010 Paris, France; audrey.baron@aphp.fr (A.B.); maud.salmona@aphp.fr (M.S.); marine.minier@aphp.fr (M.M.); audrey.gabassi@aphp.fr (A.G.); constance.delaugerre@aphp.fr (C.D.); 4 Department of Infectious Diseases, Public Health Service of Amsterdam, 1018 WT Amsterdam, The Netherlands; anders.boyd@iplesp.upmc.fr; 5 INSERM U976, University of Paris, 75010 Paris, France; 6 INSERM, Institut Pierre Louis of Epidémiology and Public Health, University of Sorbonne, 75012 Paris, France; 7 INSERM U944, Biology of Emerging Viruses, University of Paris Cité, 75006 Paris, France

**Keywords:** SARS-CoV-2, neutralizing antibodies, neutralization surrogate assays

## Abstract

Background: After the global COVID-19 crisis, understanding post-infectious immunity and vaccine efficacy remains crucial. This study aims to assess anti-SARS-CoV-2 immunity through a quantitative analysis of anti-receptor-binding domain (RBD) antibodies and rapid functional testing of the neutralizing humoral response. Methods: A retrospective analysis was conducted on samples from various cohorts, including partially vaccinated, fully vaccinated, post-COVID/no-vaccination, and post-COVID/vaccination individuals with various immune-competency statuses. The anti-RBD antibodies were measured using an automated chemiluminescence assay, while the neutralizing antibodies’ (NAbs’) activity was assessed through the lateral flow ichroma COVID-19 nAb test (LFT), a surrogate neutralization assay. Results: The analysis revealed various levels of anti-RBD antibodies and seroneutralization responses across cohorts, with the post-COVID/vaccination group demonstrating the most robust protection. A correlation between anti-RBD antibodies and seroneutralization was observed, albeit with varying strength depending on the subgroup analyzed. Longitudinal assessment following natural infection showed an initial surge followed by a decline in both measures. A cutoff of 3.0 log_10_ BAU/mL was established to predict significant seroneutralization. Conclusions: The ichroma™ COVID-19 nAb test displayed high specificity and emerged as a valuable tool for monitoring anti-SARS-CoV-2 immunity. These findings contribute to understand the antibody response dynamics and underscore the potential of rapid tests in predicting protection against SARS-CoV-2 infection.

## 1. Introduction

In the wake of the global COVID-19 pandemic, understanding the post-infectious immunity and the efficacy of vaccination has become paramount in the field of clinical virology. Individuals develop a protective immunity involving the production of neutralizing antibodies (NAbs) following SARS-CoV-2 infection and/or vaccination [1]. However, the intensity and the durability of this response remain variable across individuals and situations.

Antibodies targeting the receptor-binding domain (RBD) situated at the tip of the S1 domain, which allows the virus to enter host cells via angiotensin-converting enzyme 2, are involved in the neutralization of SARS-CoV-2 [2]. These NAbs are able to block viral entry into cells and facilitate the clearance of viral particles through Fc-mediated effector functions [3]. Despite a wide range of antibody responses during infection, only a small fraction of these antibodies display neutralizing abilities [4], and accurately quantifying NAb activity remains challenging in predicting the effective neutralization of SARS-CoV-2 [5,6,7]. This led to the development of functional neutralization assays that quantify the effective ability of sera antibodies to inhibit either in vitro infection of sensitive cells by SARS-CoV-2 (virus neutralization test, VNT) or interactions of RBD domain spike protein-targeting antibodies to the cell receptor ACE-2 (surrogate neutralization assays, sVNTs). While VNT, the gold standard, is time-consuming and biohazardous, reliable sVNTs are essential for large-scale studies.

Lateral flow tests (LFTs) are the most advanced sVNT systems developed to evaluate anti-SARS-CoV-2 neutralizing activity [8,9,10,11]. These assays are cost-effective, easy to perform, and give results in less than 30 min, providing the opportunity of point-of-care analysis. The LFT ichroma™ COVID-19 nAb has been proven to accurately correlate with the effective seroneutralization levels evaluated by the standard VNT [12,13]. After confirming the specificity of this assay using a broad range of sera from the pre-epidemic era, we analyzed seroneutralization levels across various populations, including infected and/or vaccinated individuals who were either immunocompromised or immunocompetent. We also analyzed the kinetics of neutralizing responses after a natural infection. We compared the results of the surrogate assay with the quantitation of anti-RBD Abs and determined anti-RBD levels that may be predictive of a neutralization activity among our study populations.

## 2. Materials and Methods

### 2.1. Study Design and Population

This study was a retrospective analysis of individual attending care and health care workers of Saint-Louis Hospital and Saint-Antoine Hospital, Paris, France (Assistance-Publique Hôpitaux de Paris), with various histories of SARS-CoV-2 vaccination and of COVID infection. Samples were drawn between 3 April 2020 and 21 June 2021 and stored at −80 °C. Written informed consent was obtained from all individuals to use their stored samples and personal data for non-interventional research.

We analyzed the neutralization response of five different groups of individuals: (1) immunocompetent health care workers who received partial COVID-19 vaccination, i.e., receiving only one dose of the ChAdOx1-nCov19 (Astra Zeneca, *n* = 27, BNT162b2 vaccine, *n* = 40), defined as “part-VAC”; (2) immunocompetent health workers who received complete COVID-19 vaccination, i.e., receiving either two doses of the BNT162b2 vaccine (*n* = 58) or one dose of ChAdOx1-nCov19 and then one dose of the BNT162b2 vaccine (*n* = 9), defined as “full-VAC”; (3) immunocompromised individuals seeking care at the Hematology Department and dialyzed immunocompromised patients waiting for “naive” renal transplant who received complete COVID-19 vaccination, i.e., two doses of the BNT162b2 vaccine (*n* = 156) defined as “IC/full-VAC”; (4) immunocompetent non-vaccinated individuals with a previous SARS-CoV-2 infection (documented by a positive RT-PCR test result), defined as “post-COVID/no-VAC”; and (5) immunocompetent individuals who received at least one COVID-19 vaccine dose of either ChAdOx1-nCov19 vaccine (*n* = 15), or BNT162b2 vaccine (*n* = 16) or mRNA1273 vaccine (*n* = 12), after a previous SARS-CoV-2 infection, defined as “post-COVID/VAC”.

For most individuals belonging to the vaccinated groups, two serological assays were realized: one on day 28 after the first dose and another one 14 days after the second vaccine dose (day 42).

For a subset of 20 individuals in the post-COVID/no-VAC group, samples were collected at regular intervals (i.e., day 0 of symptoms, every 4 days until day 28, and on day 60) after the first PCR positivity.

### 2.2. Serological Assays

Immunogenicity was assessed by an automated chemiluminescence assay, using SARS-CoV-2 IgG II Quant assay (Abbott, Rungis, France) for the quantitative detection of anti-S IgG (directed at the SARS-CoV-2 S protein). We also used the SARS-CoV-2 assay (Abbott, Rungis, France) for the qualitative detection of anti-N IgG (directed at the SARS-CoV-2 N protein), to monitor the humoral response against natural infection. All markers were assessed on the AlinityI platform (Abbott, Rungis, France) according to the manufacturer’s instructions.

### 2.3. Neutralization Testing

A SARS-CoV-2 surrogate neutralization assay, based on the interaction with the antibody-mediated blockade of the ACE-2-S protein, was used according to the manufacturer’s instructions (ichromax COVID-19 nAb, Boditech, Republic of Korea). Briefly, the SARS-CoV-2 NAbs present in sera samples were preincubated with a fluorescence-labeled SARS-CoV-2 S RBD antigen, in a detection buffer containing an ACE-2–biotin conjugate. The mixture was then loaded into a lateral flow nitrocellulose matrix in which the covalent RBD–ACE-2–biotin complexes are immobilized on the streptavidin capture “Test line”. This semi-quantitative assay both correlates with a neutralizing SARS-CoV-2 Ab ELISA assay (Boditech Package insert) and a whole-virus neutralization assay in cell culture [12,13]. A fluorescence inhibition above 30% in the LFT correlates with significant neutralizing responses in standard assays and was thus considered positive by the provider.].

### 2.4. Cross-Reactivity and Specificity

To determine the specificity of the sVNT used, 184 pre-epidemic serum samples collected from patients suffering from various conditions, including acute viral diseases, were tested with the ichromax COVID-19 nAb assay.

### 2.5. Statistical Analysis

We modeled levels of the anti-RBD antibody (as log_10_-transformed BAU/mL) and seroneutralization (%) using tobit regression truncated at the lower limit of detection for the anti-RBD assay (i.e., 0.01 BAU/mL) and at the lower and upper bounds for the seroneutralization assay (i.e., 0 and 100, respectively). To account for between-individual variability arising from repeated measures, we added a random intercept to the model. The mean levels between groups were compared by adding group as a covariate to the model and testing for differences using a Wald χ^2^ test. The predicted levels along with their 95% confidence intervals (CIs) were obtained from the model.

To determine the relationship between anti-RBD antibody and seroneutralization levels, we first used the repeated measures correlation coefficient developed by Bland and Altman [14], which accounts for variation from repeated measurements within individuals. This coefficient was calculated both overall and stratified on group using the “rmcorr” command in STATA [15]. We then examined the kinetics of anti-RBD antibody and seroneutralization levels, separately, in the subgroup of individuals with longitudinal samples obtained after the start of symptoms. We modeled the level of anti-RBD antibody or seroneutralization, *Y*, over time, *t*, as follows:

$$Y\left(t\right)=\left\{\begin{array}{cc} \frac{a}{1+e^{\left(-k(t-x_{0}\right)}}, & t\leq 20\\ b_{0}+b_{1}t, & t>20 \end{array}\right.$$

where the first 20 days were modeled as a logistic growth curve function with maximum value *a*, logistic growth rate of *k*, and the value of *t* at the sigmoid midpoint *x*_0_. After 20 days, *Y*(*t*) was modeled as a linear function with the intercept *b*_0_ and a linear change in the slope *b*_1_. The model was estimated using the “nl” command in STATA, while accounting for repeated measures within individuals using a cluster variance estimator.

To determine the capacity of anti-RBD antibody levels to predict seroneutralization, defined as a level > 30% (ichromax COVID-19 nAb, Boditech, Republic of Korea), we modeled the probability of seroneutralization at levels of anti-RBD antibody using logistic regression. We, again, added a random intercept to the model to account for repeated measures within individuals. We calculated the area under the receiving operator characteristic (AUROC) curve for continuous anti-RBD antibody levels. We then determined the sensitivity (Se), specificity (Sp), positive and negative predictive values (PPV and NPV, respectively), and positive and negative likelihood ratios (LR+ and LR−, respectively) of several cutoffs of anti-RBD antibodies to predict seroneutralization.

All the statistical analyses were carried out using STATA (v15.1, College Station, TX, USA) and significance was determined at *p* < 0.05.

## 3. Results

### 3.1. Description of the Study Population

In total, 618 samples were collected from 386 individuals. Of these samples, 65 (10.5%) were from the part-VAC group (64 individuals), 76 (12.3%) from the full-VAC group (67 individuals), 156 (25.2%) from the IC/full-VAC group (156 individuals), 277 (44.8%) from the post-COVID/no-VAC group (96 individuals), and 44 (7.1%) from the post-COVID/VAC group (43 individuals). Of note, 40/386 (10.4%) individuals contributed samples to two groups.

The characteristics of the study population and sampling are compared between groups in Table 1. The median age was highest in the full-VAC group [68 years, interquartile range (IQR) = 58–78], followed by 53 years (IQR =38–56) in both the post-COVID/no-VAC and post-COVID/VAC groups and was lowest in the part-VAC and full-VAC groups (42 years, IQR = 38–56). Four-fifths of the samples from the part-VAC, full-VAC, and post-COVID/VAC groups were from females, while the majority of samples from the IC/full-VAC and post-COVID/no-VAC groups were from males.

For individuals with COVID-19, the median time from COVID-19 symptom onset and sampling was 302 days longer in the post-COVID/VAC group compared to that in the post-COVID/no-VAC group (*p* < 0.0001). For the samples from vaccinated individuals, the median time since last vaccination was slightly longer (9 days) in the part-VAC and post-COVID/VAC group compared to that in both full-VAC groups (with or without immunocompromised individuals). Most individuals from these groups received either the ChAdOx1-nCov19 and/or the BNT162b2 vaccine, yet all individuals in the IC/full-VAC group received the BNT162b2 vaccine (Table 1).

### 3.2. Specificity of the Surrogate Seroneutralization Marker

To assess the specificity of the sVNT assay, 184 pre-pandemic serum samples were analyzed. Weak positivity (31.1 to 54.7% inhibition) was observed in six samples, yielding an assay specificity of 96.7% (95%CI: 94.1-99.31). Four of these samples were collected during other acute viral infections (one primary EBV, two with acute HAV, and one with acute HEV infections). Additionally, one sample was from a patient with a positive rheumatoid factor, and another from a patient with a chronic HCV infection. The complete results of the specificity analysis are detailed in the Supplementary data.

### 3.3. Differences in Antibody and Seroneutralization Levels between Groups

**Figure 1:**
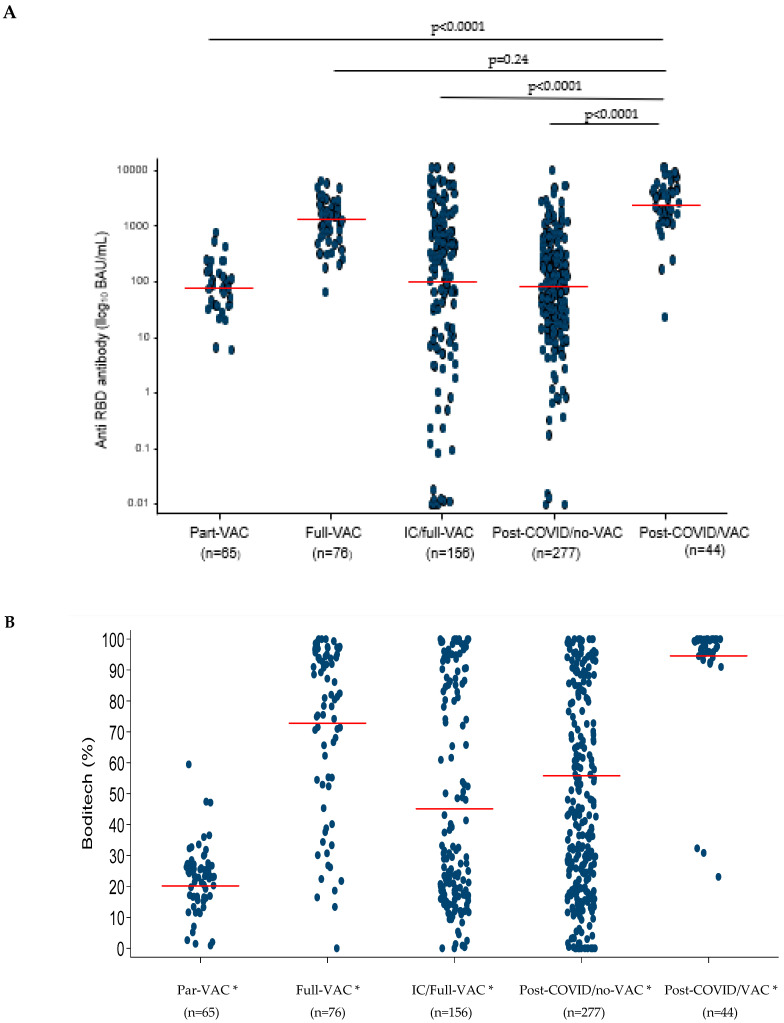
Anti-RBD antibody and seroneutralization levels in the various study groups. Data represent 618 samples collected from 386 individuals. Mean (red line) and individual (blue dots) levels of anti-RBD antibody (log_10_-transformed) and seroneutralization (Boditech %) are given in (**A**) and (**B**), respectively. Groups are defined as individuals who received partial vaccination (part-VAC), full vaccination (full-VAC), immunocompromised individuals with full vaccination (IC/full-VAC), individuals with coronavirus disease 2019 who did not receive vaccination (post-COVID/no-VAC) or received at least one vaccine (post-COVID/VAC). * All the groups were significantly different from one another (all group-by-group comparisons, *p* < 0.006).

As shown in Figure 1A, the highest anti-RBD antibody levels were observed in samples from the post-COVID/VAC group (2366.0 BAU/mL, 95%CI = 564.6–4167.4) and from the full-VAC (1325.4 BAU/mL, 95%CI = 548.4–2102.5) (difference with post-COVID/VAC group, *p* = 0.24).

Steeply lower levels of anti-RBD antibody were observed in the IC/full-VAC (102.9 BAU/mL, 95%CI = 61.0–144.7), post-COVID/no-VAC (85.0 BAU/mL, 95%CI = 45.2–124.9), and part-VAC groups (77.6 BAU/mL, 95%CI = 30.6–124.7). The differences in each of these groups compared to samples from the post-COVID/VAC group are significant (*p* < 0.0001).

Similarly, as shown in Figure 1B, the highest mean % seroneutralization was observed in samples from the post-COVID/VAC and full-VAC groups (94.6, 95%CI = 85.6–100 and 72.8, 95%CI = 66.0–79.6, respectively). A lower mean % seroneutralization was found in samples from the IC/full-VAC and post-COVID/no-VAC groups (45.2, 95%CI = 40.3–50.0 and 55.8, 95%CI = 50.1–61.4, respectively). For this last group, a significant decline in seroneutralization levels was observed for patients tested more than 4 months after the disease (median 36%, 75%CI: 27–75), compared to those tested earlier (median 89%, 75%CI: 47–97, *p* < 0.001) (see Table S1 and Figure S1 in the Supplemental Materials). Samples from the part-VAC group had the lowest mean % seroneutralization (20.2, 95%CI = 13.1–27.2). All the groups were significantly different from one another (all group-by-group comparisons, *p* < 0.005).

### 3.4. Relationship between Seroneutralization and Antibody Levels

#### 3.4.1. Seroneutralization Levels as a Function of Antibody Levels

**Figure 2:**
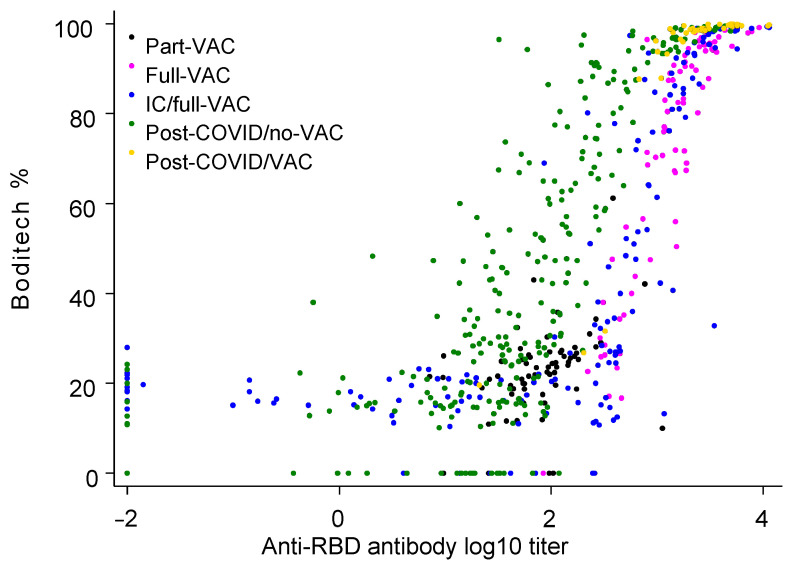
Anti-RBD antibody and seroneutralization in the entire study population. Data represent 618 samples collected from 386 individuals. Levels of anti-RBD antibody (log_10_-transformed) are plotted against seroneutralization levels (Boditech %) for each individual. Colors highlight groups of immunocompetent individuals who received partial vaccination (part-VAC), full vaccination (full-VAC), immunocompromised individuals with full vaccination (IC/full-VAC), individuals with coronavirus disease 2019 who did not receive vaccination (post-COVID/no-VAC) or received at least one vaccine (post-COVID/VAC).

The repeated measures correlation between anti-RBD antibody and seroneutralization levels was 0.627 (*p* < 0.0001). Importantly, this correlation was strongest in samples from the full-VAC and post-COVID/VAC groups (0.916, *p* = 0.0002 and 0.830, *p* < 0.0001) and lower in the IC/full-VAC (0.669, *p* < 0.0001), post-COVID/no-VAC (0.539, *p* < 0.0001), and part-VAC (0.329, *p* = 0.007) groups. The levels of anti-RBD antibodies and seroneutralization for each sample are plotted in Figure 2, while stratified on groups. From this figure, the seroneutralization % appears to increase as a logistic function with increasing levels of anti-RBD antibody levels.

#### 3.4.2. Longitudinal Changes in Antibody and Seroneutralization Levels after Natural Infection

**Figure 3:**
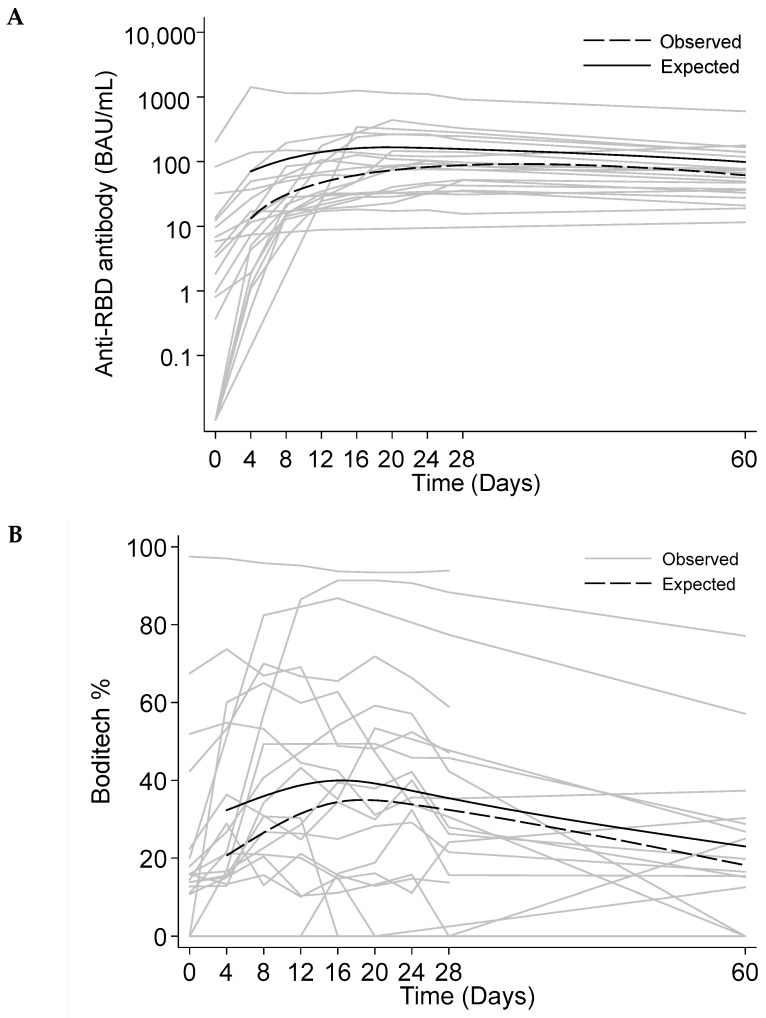
Kinetics of anti-RBD antibody and seroneutralization levels after natural infection. This dataset comprises 168 samples collected longitudinally from 20 individuals post-infection. Individual trajectories for anti-receptor-binding domain (RBD) antibody and seroneutralization (Boditech) levels are given in (**A**,**B**), respectively. Day 0 is defined as the first day of COVID symptoms, reported by the patients. For both graphs, the median spline of observed and predicted levels over time are provided as “observed” and “expected” curves. Predicted values were obtained from a non-linear, bi-phasic, growth–decay model described in the statistical analysis.

In the subgroup of individuals with natural infection, we longitudinally measured the levels of anti-RBD antibodies and seroneutralization and modeled the kinetics of both parameters, depicted in Figure 3A (anti-RBD antibodies) and Figure 3B (seroneutralization). The seroneutralization levels exhibited a much wider variation over time than the anti-RBD antibody levels (Figure 3A,B). Based on the parameters of the kinetic model, both anti-RBD antibodies and seroneutralization levels demonstrated rapid exponential growth in the first 20 days after infection (fastest growth rate at 0.222, 95%CI = 0.088–0.357 and 0.313, 95%CI = 0.074–0.552, respectively). After 20 days post-infection, we observed a non-significant decline in anti-RBD antibodies (−1.8 BAU/mL per day, 95%CI = −3.6–0.1, *p* = 0.063), while seroneutralization levels significantly decreased 0.40% per day, (95%CI = −0.73–−0.06, *p* = 0.023).

### 3.5. Antibody Levels Predicting Seroneutralization

**Figure 4:**
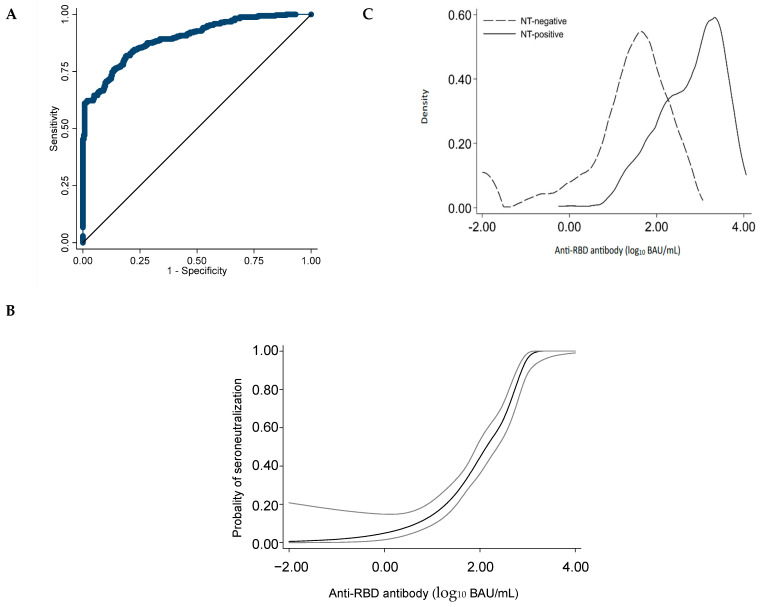
Correlation between anti-RBD antibody and seroneutralization. Data represent 618 samples collected from 386 individuals. The receiving characteristic curve of anti-receptor-binding domain (RBD) antibody levels to predict seroneutralization is given in (**A**). The probability of seroneutralization is modeled across levels of anti-RBD antibodies in (**B**), while gray bands around the black line represent the 95% confidence interval. The density histograms of anti-RBD antibody levels are provided for individuals with or without seroneutralization (NT) in (**C**). For these analyses, all anti-RBD antibody levels were log_10_-transformed.

The AUROC of the continuous anti-RBD antibody levels to predict seroneutralization was 0.895 (95%CI = 0.871–0.919) (Figure 4A), demonstrating a rather high predictive capacity. The probability of attaining significant seroneutralization with increasing levels of anti-RBD antibody levels is shown in Figure 4B. This probability quickly increases between 2 and 2.5 log_10_ BAU/mL, reaching 1 at levels above 3.0 log_10_ BAU/mL. However, below 2 log_10_ BAU/mL, it is difficult to predict the presence or absence of seroneutralization, with some individuals with low anti-RBD levels, which still displays a seroneutralization activity. The distributions of anti-RBD antibody levels are shown between individuals with and without seroneutralization in Figure 4C, demonstrating that the two distributions meet between 1.5 and 2.5 log_10_ BAU/mL.

As shown in Table 2, the Se, NPV, and LR− to predict seroneutralization all declined when increasing the cutoff of the anti-RBD antibodies. The opposite was the case for Sp, PPV, and LR+. The rather high distribution of seroneutralization levels in some groups (i.e., full-VAC and post-COVID/VAC) precluded analysis stratified on groups.

## 4. Discussion

The neutralizing capacity of antibodies plays a pivotal role in safeguarding against SARS-CoV-2 infections [6]. Therefore, accurately determining the neutralizing responses conferred by natural SARS-CoV-2 infection and/or anti-SARS-CoV-2 vaccination is essential for predicting individual protection and adapting vaccination strategies, in light of the worldwide emergence of escape variants, interindividual variability in immune response, and waning of immune protection with time. This determination relies on the quantitation of the circulating Abs directed against the spike protein of the virus (or its RBD domain) and/or functional assays quantifying the titers of neutralizing Abs able to inhibit viral infection.

Compared to crude antibody titers, which may not correlate with immune memory against SARS-CoV-2, NAb titers demonstrated better predictive value for protective immunity [16,17].

The gold standard methods for measuring NAbs are based on virus neutralization assays, using infectious particles in cell cultures. All these assays are time consuming, potentially biohazardous, and require biosafety level 3 (BSL-3) facilities. For this reason, several surrogate assays, based on the quantification of antibody-mediated blockage of ACE-2–Spike protein interactions, have been developed.

We combined the EIA quantitation of an anti-RBD and a rapid surrogate assay for seroneutralization in sera obtained from groups of individuals with various levels of immunocompetency, either vaccinated, infected by SARS-CoV-2, or both.

Our results demonstrate a strong correlation between the levels of anti-Spike Abs and seroneutralization activities as measured by the surrogate assay, in both COVID-19-recovered and -vaccinated individuals. However, the strength of this correlation varied across the studied populations, with the strongest correlation observed in the full-vaccination (full-VAC) and post-COVID/vaccination (post-COVID/VAC) groups, while it was lower in the other groups. Secondly, disparities in antibody and seroneutralization levels were identified between groups, with the post-COVID/VAC and full-VAC groups exhibiting the highest anti-RBD antibody and neutralization levels.

Longitudinal analysis showed rapid exponential growth in both anti-RBD antibodies and seroneutralization levels within the first 20 days after infection. Subsequently, a non-significant decline in anti-RBD antibodies and a significant decline in seroneutralization levels were observed. This dynamics of seroneutralization levels with time has been reported before [18] and explains why some “post-COVID” patients (especially those tested more than 4 months after the disease) do not display any significant seroneutralization activity. This wane, also reported after vaccination [19,20], explains why some fully vaccinated or “post-COVID/VAC” individuals did not show a significant seroneutralization level at the time they were tested.

Lastly, our study demonstrated that significant seroneutralization was usually achieved with levels of anti-Spike antibodies above 3.0 log_10_ BAU/mL. However, despite the linear correlation between neutralizing SARS-CoV-2 titers and anti-RBD IgG, our analysis indicated the difficulty in identifying a clear anti-RBD cutoff able to predict a significant neutralizing activity, especially in the “mid” levels between 1.5 and 2.5 log_10_ BAU/mL (Table 2 and Figure 4C).

Additionally, the effective titer for protective anti-spike antibodies against SARS-CoV-2, which has not been established yet, largely depends on the SARS-CoV-2 variant of interest [13], since the unceasing circulation of the virus leads to the emergence of novel viral sublineages, escaping previous immune responses [21].

Several studies have demonstrated significant agreement between the sVNT assays and the gold standard neutralization assay performed in culture, with correlations ranging from moderate to strong [9,10,11,22,23,24,25]. Among them, LFTs for detecting SARS-CoV-2 NAbs offer several advantages, including practicability, speed, and semi-quantitative assessment of functional inhibition. Notably, using the ichroma™ COVID-19 nAb test, the detection of NAbs in SARS-CoV-2 can be completed in only 15 min. Patients with severe SARS-CoV-2 infections have shown better correlations between antibody levels and sVNT results, corroborating previous reports of a wide range of SARS-CoV-2 NAb titers, which vary according to disease severity [7,26,27].

Our study had certain limitations. First, the delay between sampling and the onset of COVID symptoms varied significantly among the infected individuals in our panel, which may explain the broad range of neutralization responses observed in these patients. Second, we lacked data on the severity of infection, which may impact the intensity of the humoral responses. Third, we did not compare the results of the LFT with the gold standard neutralization assay. However, this comparison has been performed earlier by our team and others [12,13], confirming the strong performances of this surrogate assay and its utility in predicting the seroneutralization activity of sera.

Additionally, the LFT used in this study was developed during the first wave of SARS-CoV-2 infection and, therefore, incorporated in its cassette the RBD epitope of the alpha variant of SARS-CoV-2 that circulated at that moment. Other variants have emerged worldwide since then, and the most prevalent ones display substitutions and/or deletions in this domain that result in significant escape from post-vaccinal and/or post-infection immunity [28]. As a result, some studies determined, for this sVNT assay, higher values of the inhibition cutoff to predict effective neutralization against the more resistant Delta and Omicron variants [12,13]. Nevertheless, all the samples analyzed in our study were collected during the first wave of COVID-19 in France (from April 2020 to June 2021), during which the alpha variant was predominantly circulating, accounting for over 86% of cases [29].

Finally, this test does not assess ACE-2-independent entry pathways of SARS-CoV-2 into susceptible cells or non-antibody-mediated immunity against SARS-CoV-2 [30], but these mechanisms are of less importance compared to the immune protection conferred by neutralizing antibodies against ACE-2–Spike interactions.

Despite these limitations, surrogate functional assays such as the Ichroma™ COVID-19 nAb test, with appropriate cutoffs adapted to the circulating viruses, proved to be a valuable tool for monitoring anti-SARS-CoV-2 immunization and should be useful, in addition to the quantitation of anti-RBD spike Abs, to monitor post-infectious or post-vaccine protection, especially in individuals with low or intermediate levels of antibodies.

## Figures and Tables

**Table 1:** Description of the study characteristics according to samples.

Characteristic	Group					*p* *
	Part-VAC	Full-VAC	IC/Full-VAC	Post-COVID/No-VAC	Post-COVID/VAC	
Individual level ^§^	(*n* = 64)	(*n* = 67)	(*n* = 156)	(*n* = 96)	(*n* = 43)	
Age, years ^†^	45 (38–56)	42 (38–56)	68 ^a^ (58–78)	57 (34–67)	54 (34–67)	<0.0001 ^a^
Male/female (% male) ^‡^	6/20 (23)	8/34 (19)	95/61 (61) ^b^	46/22 (68) ^b^	4/15 (21)	<0.0001 ^b^
Sample level	(*n* = 65)	(*n* = 76)	(*n* = 156)	(*n* = 277)	(*n* = 44)	
Age, years ^††^	43 (35–56)	42 (38–56)	68 ^a^ (58–78)	53 (30–67)	54 (41–62)	<0.0001 ^a^
Male/female (% male) ^‡‡^	6/21 (22)	11/40 (22)	95 ^b^/61 (61)	168 ^b^/39 (81)	4/16 (21)	<0.0001 ^b^
Days since start of COVID-19 symptoms ^^^	-	-	-	25 (16–56) ^c^	327(227–359) ^c^	<0.0001 ^c^
Days since last vaccination ^¶^	27 ^d^ (23–29)	18 (8–38)	18 (15–26)	-	27 ^d^ (20–28)	<0.0001 ^d^
Vaccination						Ntp
ChAdOx1-nCov19	26	67	0	-	15	
BNT162b2	39	0	156	-	15	
ChAdOx1-nCov19 and BNT162b2	0	9	0	-	0	
Not specified	0	0	0	-	14	

Data represent 618 samples collected from 386 individuals; 40 individuals contributed samples to two groups. Median (interquartile range) or n (%) reported unless stated otherwise. Definitions of groups provided in the Section 2.1. * Groups were compared using Pearson’s χ^2^ test for categorical variables or Kruskal–Wallis test for continuous variables. ntp, no test performed. These differences are indicated by *p*-values less than 0.0001, showing that the observed differences are statistically significant: ^a^ IC/full-VAC was significantly older than the other groups. ^b^ IC/full-VAC and post-COVID/no-VAC had higher proportions of males. ^c^ Post-COVID/no-VAC and post-COVID/VAC differed significantly. ^d^ Significant differences between part-VAC and post-COVID/VAC with the other vaccinated groups. ^§^ For individuals contributing repeated samples in the same group, data from the first observation were considered. ^†^ Missing data: part-VAC, *n* = 38; full-VAC, *n* = 25; post-COVID/no-VAC, *n* = 28; post-COVID/VAC, *n* = 24. ^‡^ Missing data: part-VAC, *n* = 38; full-VAC, *n* = 25; post-COVID/no-VAC, *n* = 40; post-COVID/VAC, *n* = 24. ^††^ Missing data: part-VAC, *n* = 38; full-VAC, *n* = 25; post-COVID/no-VAC, *n* = 70; post-COVID/VAC, *n* = 24. ^‡‡^ Missing data: part-VAC, *n* = 38; full-VAC, *n* = 25; post-COVID/no-VAC, *n* = 176; post-COVID/VAC, *n* = 24. ^^^ Missing data: post-COVID/no-VAC, *n* = 4; post-COVID/VAC, *n* = 23. ^¶^ Missing data: part-VAC, *n* = 1; full-VAC, *n* = 2; post-COVID/VAC, *n* = 7. Abbreviations: COVID-19, coronavirus disease 2019.

**Table 2:** Evaluating anti-RBD antibody levels as a predictor of seroneutralization.

Anti-RBD Cutoff (BAU/mL)	Seroneutralization		Classification Probabilities					
	Pos	Neg	Se	Sp	PPV	NPV	LR+	LR−
			93.9	47.4	69.8	85.7	1.78	0.129
≥1.5 log_10_	323	140						
<1.5 log_10_	21	126						
			84.0	77.4	82.8	78.9	3.72	0.206
≥2.0 log_10_	289	60						
<2.0 log_10_	55	206						
			66.3	92.5	91.9	68.0	8.82	0.365
≥2.5 log_10_	228	20						
<2.5 log_10_	116	246						
			48.3	99.2	98.8	59.7	64.2	0.521
≥3.0 log_10_	166	2						
<3.0 log_10_	178	264						
			15.4	100	100	47.8	∞	0.846
≥3.5 log_10_	53	0						
<3.5 log_10_	291	266						

Data represent 618 samples collected from 386 individuals. Abbreviations: RBD, receptor-binding domain; Neg, negative; Se, sensitivity; Sp, specificity; PPV, positive predictive value; NPV, negative predictive value; LR+, positive likelihood ratio; LR−, negative likelihood ratio.

## Data Availability

No new data were created or analyzed in this study. Data sharing is not applicable to this article.

## Supplementary Materials

The following supporting information can be downloaded at https://www.mdpi.com/article/10.3390/life14070791/s1: Figure S1: Anti-RBD antibody (BAU/mL) and seroneutralization (Boditech) % levels in the post-COVID/no-VAC group according time (4 month) after the disease; Table S1: Anti-RBD antibody (BAU/mL) and seroneutralization (Boditech) % levels in the post-COVID/no-VAC group according time (4 month) after the disease.
