# Supplementary material for: Anti-SARS-CoV-2 Neutralizing Responses in Various Populations: Use of a Rapid Surrogate Lateral Flow Assay and Correlations with Anti-RBD Antibody Levels
Source: Life (Basel). 2024 Jun 22;14(7):791. doi: 10.3390/life14070791 (PMC11277712; doi:10.3390/life14070791)
Supplement: Supplementary file 1 [file life-14-00791-s001.zip › life-3008285-supplementary.pdf]

Table S1: Anti-RBD antibody (BAU/mL) and seroneutralization (Boditech) % levels in the post-COVID/no-VAC group according time (4 month) after the disease

	Post-Covid/No-Vac <4months<br>*N = 65*[1]	***Post-Covid/No-Vac >4months***<br>*, N = 40*[1]	***p-value***[2]
Boditech (%)	89 (47, 97)	36 (27, 75)	<0.001
Anti-RBD antibody (BAU/ml)	2,755 (244, 10,636)	487 (232, 1,560)	0.006

[1] *Median (IQR)*

[2] *Wilcoxon rank sum test*

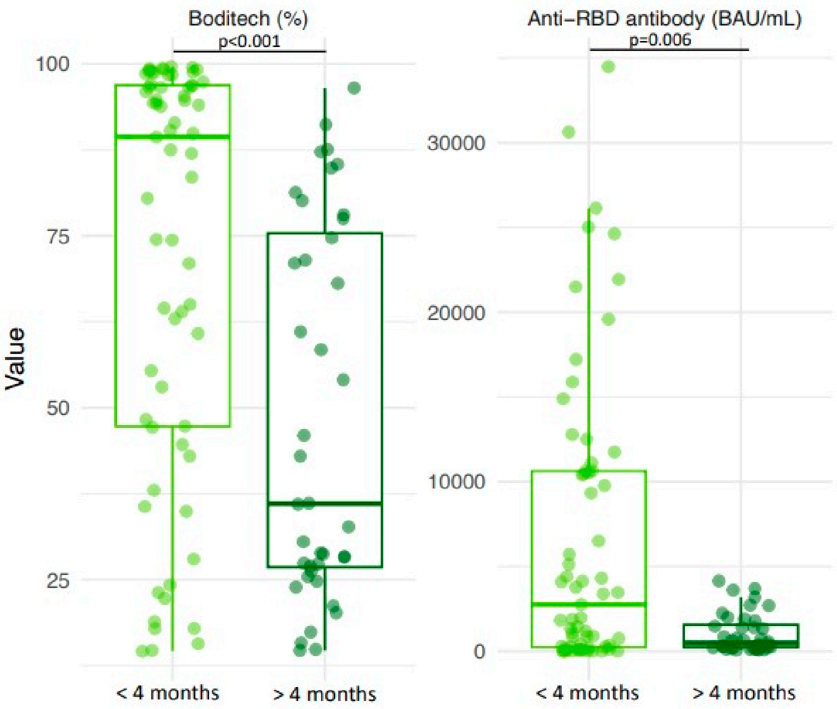

Figure S1 : Anti-RBD antibody (BAU/mL) and seroneutralization (Boditech) % levels in the post-COVID/no-VAC group according time (4 month) after the disease
